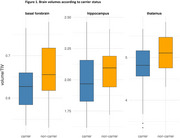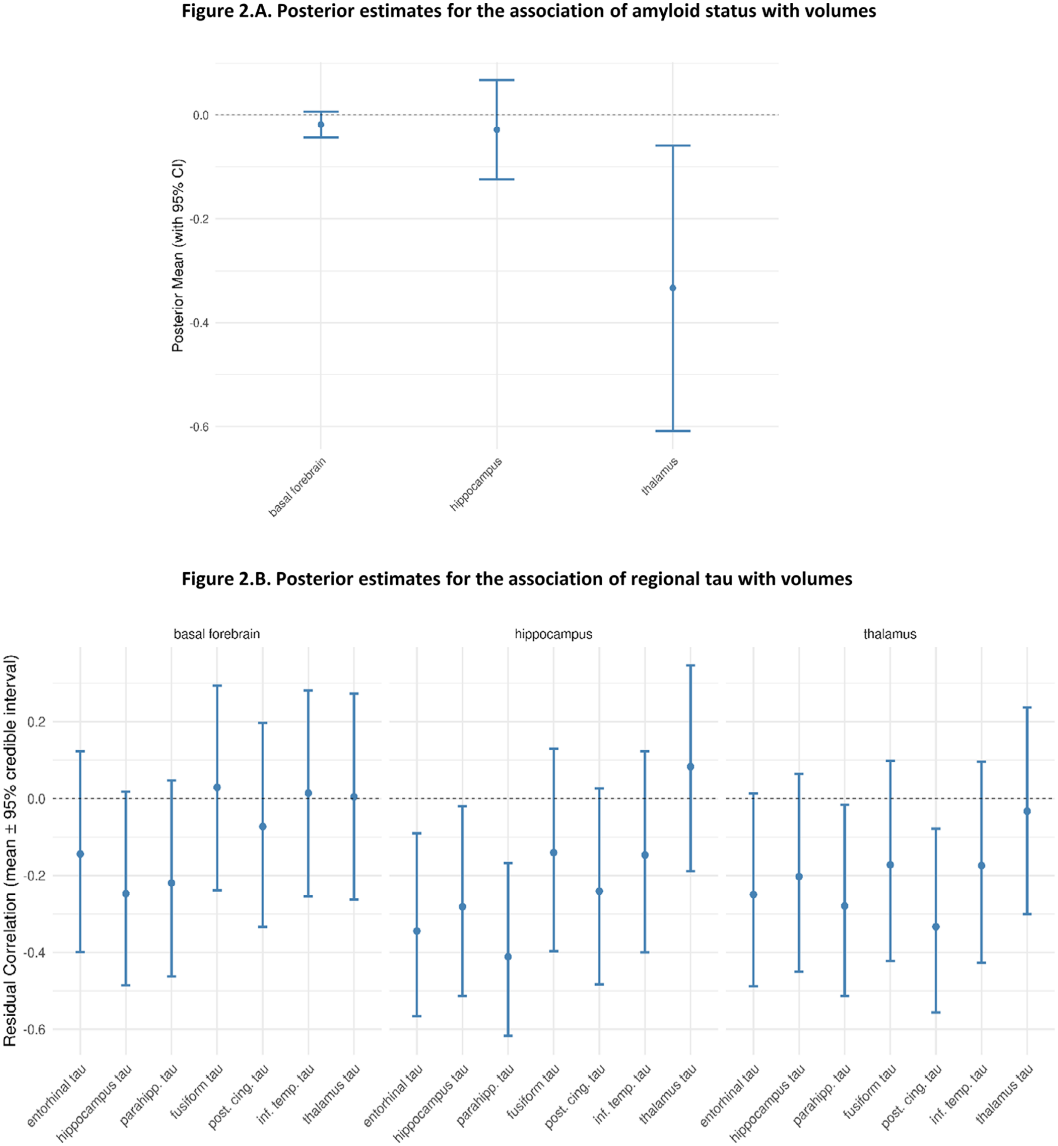# Thalamus but not basal forebrain and hippocampus atrophy in prodromal carriers of the Columbian mutation for autosomal dominant Alzheimer’s disease – a Bayesian confirmatory analysis

**DOI:** 10.1002/alz70861_108535

**Published:** 2025-12-23

**Authors:** Stefan J. Teipel, Ana Y Baena, Bing He, Lusiana Martinez, David Fernando Aguillón Niño, Yakeel T. Quiroz, Alice Grazia

**Affiliations:** ^1^ Department of Psychosomatic Medicine, Rostock University Medical Center, Rostock Germany; ^2^ German Center for Neurodegenerative Diseases (DZNE), Rostock Germany; ^3^ Grupo de Neurociencias de Antioquia, Facultad de Medicina, Universidad de Antioquia, Medellín Colombia; ^4^ Massachusetts General Hospital, Harvard Medical School, Boston, MA USA; ^5^ Grupo de Neurociencias de Antioquia, Universidad de Antioquia, Medellín, Antioquia Colombia; ^6^ Grupo de Neurociencias de Antioquia, University of Antioquia, Colombia, Medellín, Antioquia Colombia; ^7^ Deutsches Zentrum für Neurodegenerative Erkrankungen e. V. (DZNE), Rostock/Greifswald, Rostock Germany; ^8^ Department of Psychosomatic Medicine, University Medicine Rostock, Rostock, Germany, Rostock Germany

## Abstract

**Background:**

In sporadic Alzheimer's Disease (AD), the cholinergic basal forebrain degenerates early, but seems to be preserved in the Colombian *PSEN1*‐E280A genetic mutation for familial AD. In two cross‐sectional studies analyzing basal forebrain volume and connectivity from the API Colombia cohort, we found no reduction in basal forebrain volume or connectivity in carriers, but observed reduced thalamic volume. Confirming these findings in other groups with the same genetic mutation, such as those in the Colombia‐Boston (COLBOS) biomarker study, will help determine whether the new hypothesis about basal forebrain preservation in familial AD holds true.

**Method:**

We used multi‐modal imaging (MRI, amyloid and tau‐PET) data of 57 cognitively unimpaired individuals from the COLBOS study comprising *PSEN1*‐E280A carriers (n = 27) and non‐carriers (n =30). Staging of functional impairment was done using the Functional Assessment Staging Tool (FAST). Volumetry analyses were performed using basal forebrain, hippocampus and thalamus as regions of interest. We used Bayesian multiple regression with both flat and informed priors to test the hypothesis of preservation. As a secondary analysis, we investigated the associations of amyloid and tau pathology with brain volumes.

**Result:**

We found evidence for no effect of mutation carrier status on basal forebrain volume (BF₁₀: flat = 0.076; inf =0.54) and hippocampal volume (BF₁₀: flat =0.16; inf =1.05). In contrast, we observed moderate evidence for an effect of mutation carrier status on thalamus volume, with smaller volumes in mutation carriers (BF₁₀: flat =5.42; inf =8.74) (Figure 1). Lastly, we found strong evidence against an effect of amyloid status on basal forebrain and hippocampus, but moderate evidence for an effect on thalamic volume (Figure 2A). Similarly, tau pathology was not associated with basal forebrain, but showed associations with thalamus and hippocampus. (Figure 2B).

**Conclusion:**

Our findings show that in the *PSEN1*‐E280A cohort, the cholinergic basal forebrain and hippocampus are not affected early in the disease, whereas the thalamus is, pointing to important considerations for treatment target selection in this group. These results should be further explored in other autosomal dominant AD mutations.